# ^1^H-NMR Based Serum Metabolomics Highlights Different Specific Biomarkers between Early and Advanced Hepatocellular Carcinoma Stages

**DOI:** 10.3390/cancers12010241

**Published:** 2020-01-18

**Authors:** Andrea Casadei-Gardini, Laura Del Coco, Giorgia Marisi, Fabio Conti, Giulia Rovesti, Paola Ulivi, Matteo Canale, Giovanni Luca Frassineti, Francesco Giuseppe Foschi, Serena Longo, Francesco Paolo Fanizzi, Anna Maria Giudetti

**Affiliations:** 1 Division of Medical Oncology, Department of Medical and Surgical Sciences for Children and Adults, University Hospital of Modena, 41125 Modena, Italy; casadeigardini@gmail.com (A.C.-G.); giulia.rovesti@gmail.com (G.R.); 2 Department of Biological and Environmental Sciences and Technologies, University of Salento, 73100 Lecce, Italy; laura.delcoco@unisalento.it (L.D.C.); serena.longo@unisalento.it (S.L.); anna.giudetti@unisalento.it (A.M.G.); 3 Biosciences Laboratory, Istituto Scientifico Romagnolo per lo Studio e la Cura dei Tumori (IRST) IRCCS, 47014 Meldola, Italy; giorgia.marisi@irst.emr.it (G.M.); paola.ulivi@irst.emr.it (P.U.); matteo.canale@irst.emr.it (M.C.); 4 Department of Internal Medicine, Degli Infermi Hospital, 48018 Faenza, Italy; fabio.conti2@studio.unibo.it (F.C.); fgfoschi@gmail.com (F.G.F.); 5 Department of Medical Oncology, Istituto Scientifico Romagnolo per lo Studio e la Cura dei Tumori (IRST) IRCCS, 47014 Meldola, Italy; luca.frassineti@irst.emr.it

**Keywords:** hepatocellular carcinoma, NMR, metabolomics, OPLS-DA, radiofrequency, sorafenib

## Abstract

The application of non-targeted serum metabolomics profiling represents a noninvasive tool to identify new clinical biomarkers and to provide early diagnostic differentiation, and insight into the pathological mechanisms underlying hepatocellular carcinoma (HCC) progression. In this study, we used proton Nuclear Magnetic Resonance (^1^H-NMR) Spectroscopy and multivariate data analysis to profile the serum metabolome of 64 HCC patients, in early (n = 28) and advanced (n = 36) disease stages. We found that ^1^H-NMR metabolomics profiling could discriminate early from advanced HCC patients with a cross-validated accuracy close to 100%. Orthogonal partial least squares discriminant analysis (OPLS-DA) showed significant changes in serum glucose, lactate, lipids and some amino acids, such as alanine, glutamine, 1-methylhistidine, lysine and valine levels between advanced and early HCC patients. Moreover, in early HCC patients, Kaplan–Meier analysis highlighted the serum tyrosine level as a predictor for overall survival (OS). Overall, our analysis identified a set of metabolites with possible clinical and biological implication in HCC pathophysiology.

## 1. Introduction

Hepatocellular carcinoma (HCC) is one of the leading causes of cancer-related deaths worldwide. The disease displays a complex molecular landscape that hampers patient’s prognosis and therapy [1]. HCC commonly arises in people with underlying liver diseases associated to viral infections (chronic hepatitis B and C), toxic (alcohol and aflatoxin), metabolic (diabetes, hemochromatosis, and non-alcoholic fatty liver disease) and immune (autoimmune hepatitis and primary biliary) factors [1]. 

Although many therapeutic approaches have so far been established for HCC treatment, the unavailability of adequate biomarkers for early HCC diagnosis causes a poor prognosis. Hence, specific and reliable biomarkers for the early diagnosis and prognosis of HCC can improve the understanding of HCC etiology and allow an early detection of the disease for the reduction of HCC incidence [2]. Altered tumor metabolism is now considered a hallmark of cancer, with diagnostic and therapeutic implications in several cancer types, including HCC [3,4]. An altered metabolic profile supports tumor growth, proliferation and survival by increasing energy production, macromolecular biosynthesis and the maintenance of redox balance [3]. Significant modifications in metabolic pathways have been identified in HCC, and associated with clinical outcomes [5]. For instance, early-stage HCC tumors with a poor overall survival rate are characterized by proteomic alterations in glycolysis and cholesterol metabolism, compared to patients with a better prognosis [5]. Metabolic alterations at the tumor level may impact systemic homeostasis, thus inducing a global metabolic reprogramming that may be useful for cancer development or progression monitoring. In the last few years, metabolite quantification by Mass Spectrometry and Nuclear Magnetic Resonance Spectroscopy approaches have been used to obtain a global, unbiased view of small molecules in biofluids and organs, thus contributing to the understanding of the molecular characteristics of many diseases. Moreover, these approaches were also used to define a series of biomarkers for early diagnoses and treatments of different diseases [6,7,8,9]. In this study, the serum of 64 HCC patients diagnosed in early (28 patients) and advanced (36 patients) disease stages was collected and profiled by the ^1^H-Nuclear Magnetic Resonance (^1^H-NMR)-based metabolomics approach coupled with multivariate data analysis. Significant differences in the serum level of 11 metabolites were identified between the two different groups of patients. Based on identified metabolites, we defined a metabolic signature useful to distinguish between HCC patients and potentially correlated with clinical characteristics.

## 2. Results

### 2.1. Patient Characteristics and Clinical Outcomes

In this study, a total of 64 patients, 28 in early (EAR) and 36 in advanced (ADV) stage were recruited. The main clinical characteristics of patients are shown in Table 1. In details, 28 EAR HCC patients were treated with radiofrequency, and included in the study in the period between February 2016 and June 2018, with a calculated median disease-free survival (DFS) of 24.5 months (3.9–27.8). The 36 ADV HCC patients were treated with sorafenib, and recruited in the study between March 2016 and June 2018, with a median overall survival (OS) of 13.1 months (95% CI 10.9–15.9). Both in EAR and particularly in ADV stages, a number of HCC patients were diabetics with only a low percentage of these being treated with metformin. In both EAR and ADV groups, most of the patients were Hepatitis C Virus (HCV) positive. Moreover, more than half of ADV HCC patients had extrahepatic diseases.

### 2.2. H-NMR Analysis of Serum Samples

**Figure 1:**
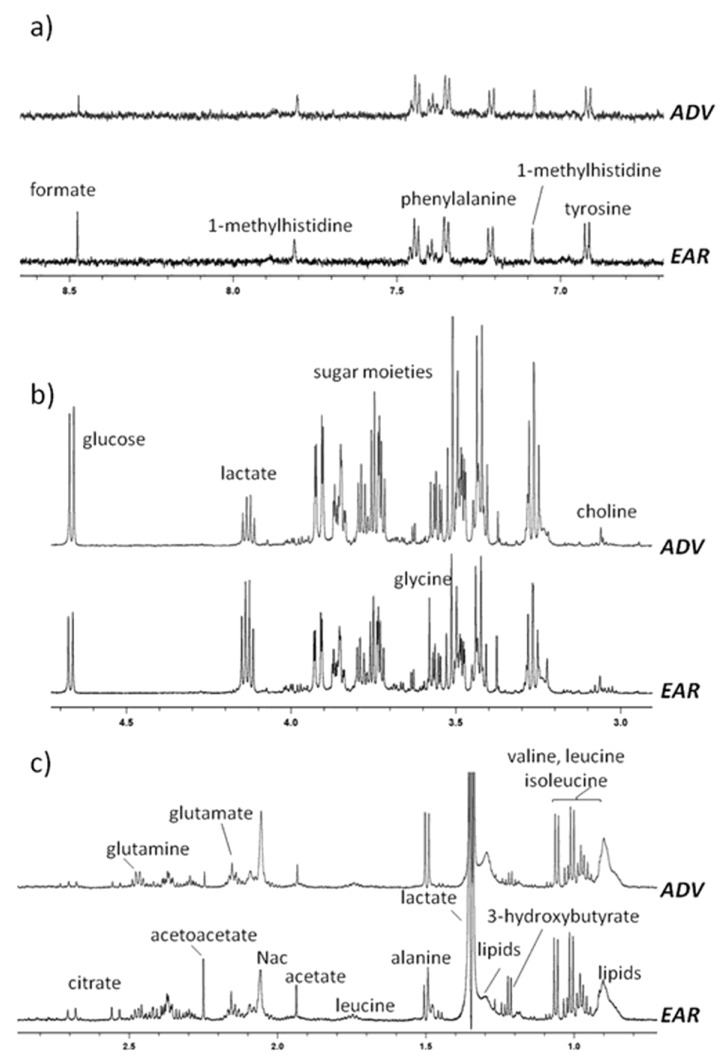
Typical proton Carr–Purcell–Meiboom–Gill nuclear magnetic resonance (^1^H CPMG NMR) spectra in the (**a**) aromatic, (**b**) sugars and (**c**) aliphatic regions, with some identified metabolites for the different groups of advanced (ADV) and early (EAR) hepatocellular carcinoma (HCC) patients referred to the specific sample spectra in the figure.

Representative proton Carr–Purcell–Meiboom–Gill nuclear magnetic resonance (^1^H CPMG NMR) spectra obtained from serum patients in the EAR and ADV HCC stages are shown in Figure 1. 2D NMR (J-resolved, homonuclear correlation spectroscopy (COSY), heteronuclear single-quantum correlation spectroscopy (HSQC), and heteronuclear multiple bond correlation spectroscopy (HMBC)) experiments were used to accurately assign the resonances of the identified metabolites, according to literature data and public databases [8,9,10,11]. The ^1^H-NMR spectra were dominated by high-intensity signals ascribable to the sugar moieties of α and β-glucose, lactate and some high molecular weight metabolites, such as lipoproteins (VLDL/LDL). Small molecules, including formate, phenylalanine, tyrosine, creatine/ phosphocreatine, histidine, 1-methylhistidine, alanine, glycine, citrate, glutamate, glutamine, methionine, acetate, succinate, valine, lactate, leucine, lysine, isoleucine, 2-hydroxyisobutyrate, 3-hydroxybutyrate and others, were also observed. Overall, no significant differences in the number of metabolites identified by ^1^H NMR in the spectra of EAR and ADV patients were observed. 

### 2.3. Multivariate Analysis of NMR Data

A multivariate statistical approach was performed by applying both unsupervised (PCA) and supervised (PLS-DA and OPLS-DA) statistical analyses on the whole NMR data. While the PCA analysis provides a general overview on the trends and patterns of data, the supervised methods need a prior knowledge of sample clustering, and are used to elucidate the most reliable class-discriminating variables for group separation [12]. At a first glance, striking differences in peak intensities emerged from a comparison between EAR and ADV HCC patients, but not depending upon HCC etiology (Figure S1) and/or extra hepatic diseases (Figure S2). 

**Figure 2:**
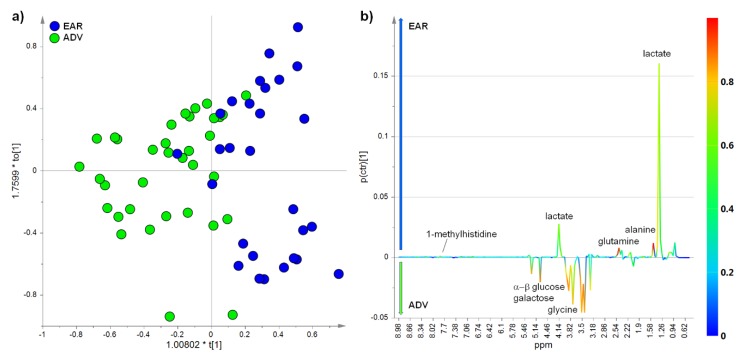
Serum metabolic profile discriminates between advanced and early HCC patients. (**a**) Orthogonal partial least squares discriminant analysis (OPLS-DA) score plot (R^2^X = 0.75, R^2^Y = 0.58, Q^2^ = 0.38 p[CV-ANOVA] = 9.00484 × 10^−5^ (Cohen’s coefficient (K) equal to 0.968, Table S1) and (**b**) the corresponding S-line plot for the model displaying the discriminant metabolites and the related predictive loadings (variables in the proton Nuclear Magnetic Resonance (^1^H-NMR) spectra. Variables are colored according to the correlation scaled loading (p(corr)). The arrows indicate the metabolite content increase for the advanced (ADV) and early (EAR) group.

The PCA model was built using the first three principal components with an explained total variance of more than 70% (R^2^X = 0.71, Q^2^ = 0.63) while the OPLS-DA model was obtained with one predictive and two orthogonal components (R^2^X = 0.75, R^2^Y = 0.58, Q^2^ = 0.38, p[CV-ANOVA] = 9.9.00484 × 10^−5^ (Cohen’s coefficient (K) equal to 0.968, Table S1) (Figure 2a). The differences in the metabolite patterns were studied by the analysis of the corresponding S-line plot (Figure 2b). From the analysis, a relative lower level of lactate (loadings corresponding to the NMR signals at 1.34 and 4.14 ppm) was observed in ADV with respect to EAR HCC patients [6]. Moreover, a relative higher level of α- and β-glucose was found in ADV with respect to EAR HCC patients. In addition to glucose, also other types of sugars, including galactose, were significantly higher in ADV HCC [13]. Alanine (NMR signal at 1.49 ppm), N-acetylglycoproteins (2.05 ppm), glycine (3.60 ppm), glutamine (2.15, 2.46 and 2.50 ppm), 1-methylhistidine (7.78 ppm) and other amino acids, such as valine and lysine, resulted in being the most discriminating metabolites between EAR and ADV HCC patients. A decreased level of alanine, glutamine, 1-methylhistidine, valine and lysine resulted in ADV with respect to EAR patients, while N-acetylglycoproteins and glycine were increased in ADV with respect to EAR samples. N-acetyl moieties of glycoproteins produced a broad resonance at 2.05 ppm in the ^1^H NMR spectrum. This signal is generally ascribable to N-acetyl protons from α1 acid glycoprotein [14]. By the integration of the corresponding selected NMR signals, a quantitative variation for discriminating metabolites between EAR and ADV HCC patients was obtained and reported in Table 2. Results, measured as the mean and standard deviation of integrals for each group, were validated by the univariate *t*-test, with an adjusted *p*-value cut-off of 0.05 [15].

### 2.4. Metabolic Pathway Analysis

**Figure 3:**
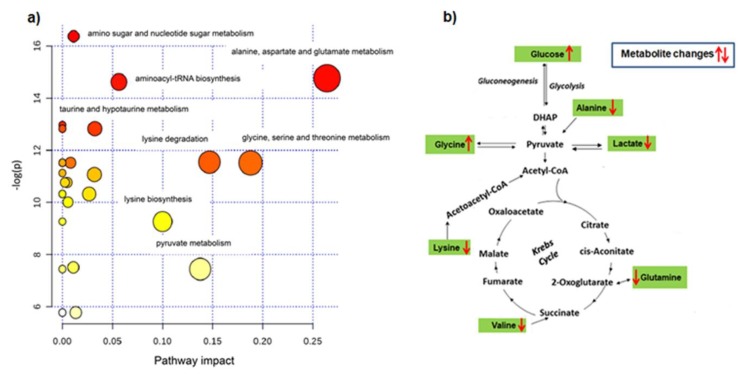
(**a**) Metabolic Pathway Analysis identifies significant differences between advanced and early HCC patients. Nodes in red indicate significance (*p* < 0.05), and the size of the nodes indicate impact. (**b**) Main pathways through which amino acids supply the Krebs cycle to furnish energy. Red arrows indicated the change direction: metabolite increased (upward arrow) and metabolite decreased (down arrow) in advanced with respect to early HCC patients.

Starting from the quantitative evaluation of discriminating metabolites between ADV and EAR HCC patients, the Metabolic Pathway Analysis was performed in order to investigate on the potential pathways that may significantly impact upon a given biological process [15,16,17]. According to both the *p*-value and the impact value, the analysis showed target pathways that could be potentially altered between the ADV and EAR HCC stages (Figure 3). Results from the pathway analysis are shown in details in Table 3, in which many pathways are tested at the same time with resulting statistical *p*-values, obtained for multiple testing. In particular, Table 3 reported the matched values over the total number of metabolites for each pathway; the original (raw *p*) and the adjusted (by Holm –Bonferroni method and FDR) *p*-value; the pathway impact value. Alanine, aspartate and glutamate metabolism, glycine, serine and threonine metabolism, lysine metabolism, aminoacyl-tRNA biosynthesis, amino sugar and nucleotide sugar metabolism, pyruvate metabolism, lysine and hypotaurine metabolism, resulted in the most relevant metabolome views potentially involved in the observed variation of EAR and ADV HCC serum metabolites, according to the *p*-value (−log(*p*)) and the impact value.

### 2.5. Kaplan-Meier Analysis of Disease-Free Survival and Overall Survival

**Figure 4:**
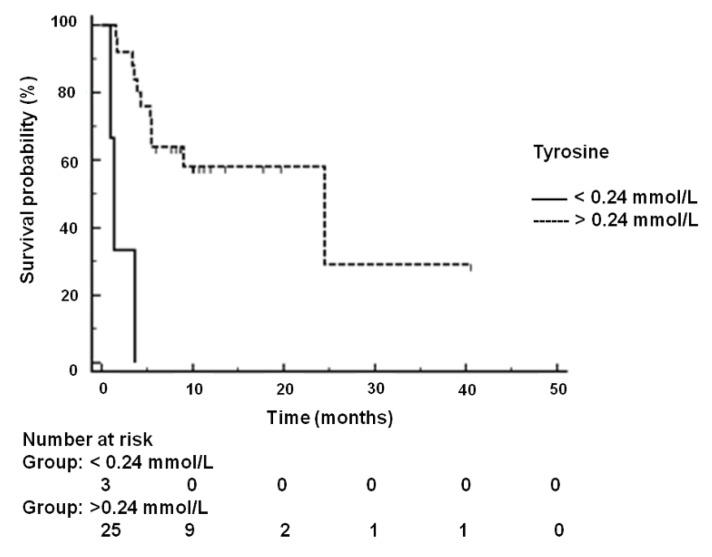
Kaplan–Meier analysis for overall survival (survival probability) for the whole 28 HCC patients in the early stage enrolled in this study.

The statistically significant differences in the metabolite profile could be also related to disease-free survival (DFS) and overall survival (OS). The capability of ^1^H-NMR profiling was therefore tested. In the cohort of EAR stage patients, at univariate analysis after the Bonferroni correction (*p* < 0.0025), the median DFS was of 24.47 months (95% CI: 5.36 to 24.470) and 1.38 months (95% CI: 1.0 to 3.6) for patients with a tyrosine value above and below an estimated threshold value of 0.24 mmol/L, directly measured in the ^1^H NMR spectrum exhibiting the specific threshold area by the standard-addition method [18] (HR = 0.01, 95% CI 0.0–0.03, *p* < 0.00001) (Figure 4). In the cohort of the ADV stage treated with sorafenib, at univariate analysis after Bonferroni correction (*p* < 0.0025) no significant correlation of the metabolite serum level with OS was found.

## 3. Discussion

During these years, the clear role of specific metabolic pathways in driving pro-tumorigenic events including tumor growth, chemoresistance and plasticity, is emerged [19,20,21,22,23,24]. An over-expression of metabolic genes regulating glycolysis, aminoacyl-tRNA biosynthesis, pyrimidine biosynthesis, purine biosynthesis and pentose phosphate pathway characterize tumor tissues compared to normal samples, thus highlighting the hypothesis of a specific metabolic signature at the tumor level [25]. Tumor mediated changes in whole-body metabolism can support the growth and proliferation, diverting key metabolites towards anabolic or catabolic processes [26]. From a clinical perspective, this underscores the opportunity to monitor systemic metabolites for studies of cancer metabolism.

This study provides a detailed snapshot of the serum metabolite profile in EAR and ADV HCC patients. The reported metabolites revealed a distinctive metabolic fingerprint in the two HCC stages. In addition, the metabolomic profiling coupled with pathway analysis provided a deeper understanding of the metabolome changes among HCC patients. Several metabolic pathways were identified, including pathways related to amino acid, pyruvate and glutamine metabolisms.

Numerous studies have reported the dysregulation of the amino acid metabolism in HCC [27,28,29,30]. Consistent with these results, we observed decreased serum levels of alanine, glutamine, 1-methylhistidine, lysine and valine in ADV with respect to EAR HCC patients; on the contrary, serum glycine level was increased in ADV vs EAR patients. These metabolite changes would be intimately associated with the progression of HCC. To note that, except for lysine and valine, which are essential amino acids, the others can be endogenously synthesized, and the liver represents, in physiological conditions, an important site of amino acid synthesis [31]. Thus, a reduced level of serum non-essential amino acids can be related to both reduced synthesis and increased utilization. Cancer cells have a high energy demand, and also require increased building blocks to sustain their rapid rate of growth, so that they plastically make adequate their metabolism to increase the utilization of alternative sources. Indeed, increased demand for amino acid has been found in malignant tumors [32]. Glutamine is an amino acid that is largely used in cancer cells, as its withdrawal from the extracellular environment can significantly affect tumor growth [33]. As known, glutamine is converted to glutamate, and further metabolized to α-ketoglutarate for ATP synthesis through the tricarboxylic acid cycle [33]. Glutamine resulted from the glutamine synthetase (GS) reaction between glutamate and ammonia in an ATP-dependent manner. Consistently, GS is a marker of HCC, and its high expression may increase the metastatic potential in HCC patients [34]. A study reported that plasma glutamine and alanine were lower in HCC patients when compared with normal subjects and patients with liver cirrhosis, indicating that the consumption of these amino acids increased in HCC [35]. Based on these observations, the lower level of both alanine and glutamine, found in the serum of ADV in comparison with EAR HCC patients, might be seen as a consequence of a higher utilization of these amino acids in the ADV HCC stage. Metabolic Pathway Analysis showed that the alanine and glutamate metabolism was the most impacting pathway differentiating EAR from ADV HCC patients. On the other hand, also the lysine degradation pathway was among the metabolisms which maximally differentiated the EAR from the ADV stage. We could therefore speculate that an increased metabolism of these amino acids (alanine, glutamate and lysine) can be considered as indicative of HCC progression. 

Interestingly, the serum glycine level positively correlated with the HCC worsening due to the higher level of this amino acid which we measured in the serum of ADV vs EAR patients. Glycine plays a key role in regulating the methylation status of cancer cells and for DNA and RNA synthesis [36]. Recently, it has been reported that a high level of blood serine/glycine might form a protective niche for the maintenance of leukemia cells in xenografted mice [37], and that a downregulation of the glycine decarboxylase, an enzyme involved in glycine catabolism, enhances HCC progression and intrahepatic metastasis [38]. Based on this knowledge, the increased serum glycine level observed in ADV vs EAR HCC patients could be seen as a result of a reduced glycine catabolism in the advanced HCC stage. Metabolic Pathway Analysis also showed that the metabolism of glycine and serine was among the most impacting processes in HCC progression. 

Valine belongs to the branched amino acids group (BCCA), for which a role in the pathophysiology of liver diseases has been established [39]. BCCA are primarily catabolized by extrahepatic tissues by a transamination reaction to form glutamate. Glutamate can be converted to glutamine by the action of the GS enzyme. Thus, decline in the level of the BCCA may affect the body glutamate–glutamine pool. Our results are consistent with these indications. Experimental evidences suggest that cancer enhances the oxidation of BCAAs and declines the BCCAs level [39]. Indeed, in advanced cirrhotic patients, it has been reported that the serum concentrations of BCCA are decreased, and the administration of BCCA-rich medicines induced positive results in patients with cirrhosis. Interestingly, valine has been demonstrated to stimulate immune response [40], thus having therapeutic potential for reducing HCC in patients with cirrhosis by restoring the immune functions [40,41,42].

We also measured a strong decrease in the 1-methylhistidine level in ADV vs EAR HCC patients. This metabolite is produced from histidine metabolism, and has been defined as a marker of skeletal muscle metabolism [43]. Histidine is an essential amino acid involved in many functions in the body; it controls gene expression and enzyme activity through methylation. The intake of histidine in a mouse model of hepatic injury has been reported to reduce the levels of inflammatory cytokines in the liver [44]. It has been reported that histidine treatment regulates hepatic glucose metabolism in type 2 diabetes [45], and improves insulin sensitivity [46]. Due to the observed higher serum glucose level in ADV with respect to EAR HCC patients, we speculated that an altered histidine metabolism, revealed by a modified serum level of 1-methylistine, might be associated with the hyperglycemia of these patients. It should be considered that about 42% of patients in the ADV HCC stage were diabetic, as evident from the clinical data reported in Table 1. Furthermore, the higher glucose level of ADV patients is in line with the increased serum amount of N-acetylglycoprotein found in ADV with respect to EAR patients. Protein glycosylation, an enzymatic process by which saccharide groups are added to the maturing proteins, is involved in fundamental molecular and cell biology processes occurring in cancer, including metastasis formation. It has also been established that especially the N-glycosite occupancy of a protein is associated with the enzymatic activity and the physical stability of glycoproteins [47], which might contribute to the metastasis of HCC.

The anaerobic metabolism of glucose generates lactate, and a high level of lactate with a low level of glucose is at the basis of the cancer glycolytic shift described as the “Warburg effect”. This metabolic shift has been found both in human and animal models of HCC [48,49,50]. Lactate is one of the most known biomarkers in tissue hypoxia and necrosis [51], and represents an important substrate for tumor energy metabolism and growth [48]. Indeed, aerobic not transformed to stromal cells can utilize lactate excreted by anaerobic tumor cells to produce pyruvate, which, in turn, can be extruded to refuel tumor cells, thus generating a pathway similar to the Cori cycle [48]. Although the serum lactate level of both EAR and ADV patients is normally higher than in control subjects (data not shown), ADV patients had lower serum lactate than EAR patients, when compared to each other. The metabolic pathway analysis assigned a high impact score (0.13756) to the pyruvate metabolism as a discriminating pathway between ADV and EAR HCC patients. Interestingly, in the same patients, a higher level of serum glucose was measured with respect to EAR patients. Based on our results, we speculate that ADV patients might have increased lactate utilization, or alternatively a decreased glucose metabolism to lactate, considering the higher serum glucose level in ADV with respect to EAR patients. This suggests that serum lactate levels may identify a metabolic classification that could improve HCC diagnosis. On the other hand, considering that increased glucose metabolism is often used as a clinical indication for cancer diagnosis, our result deserves further investigation.

Kaplan–Meier analyses which were conducted for both groups of HCC patients revealed that EAR patients with a serum tyrosine level under an estimated threshold of 0.24 mmol/L had significantly lower disease-free survival (DFS). Tyrosine is an amino acid that can be synthesized mainly in the liver from phenylalanine by the phenylalanine hydroxylase enzyme. It should be noted that the activity of phenylalanine hydroxylase was reduced in biopsies from liver cirrhosis, alcoholic hepatitis and other liver diseases [52]. 

Moreover, after oral assumption of L-phenylalanine, patients with liver cirrhosis or acute hepatitis show significant higher serum concentrations of phenylalanine, and significantly lower concentrations of tyrosine than normal persons [52]. Interestingly, it has been reported that only in the end-stage of liver disease the reaction catalyzed by phenylalanine hydroxylase may be impaired, and such defects can be corrected by transplantation [53]. On the light of these considerations, tyrosine levels can be considered an important serum biomarker for HCC progression. 

## 4. Materials and Methods

### 4.1. Patient Sampling

This was a retrospective study carried out on 64 HCC patients consecutively treated at Istituto Scientifico Romagnolo per lo Studio e la Cura dei Tumori and the Department of Internal Medicine of Faenza, from 2016 to 2018. A number of 28 patients were considered in the early (EAR) HCC stage, according with Barcelona Clinic Liver Cancer (BCLC) 0 or A, and recommended for radiofrequency, while 36 patients were diagnosed in the advanced (ADV) HCC stage, according to the American Association for the Study of Liver Diseases (AASLD) guidelines, recommended for sorafenib treatment, and refractory, or no longer amenable to locoregional therapies. EAR HCC patients had a calculated median DFS of 24.5 months (3.9–27.8), whereas ADV HCC patients had a median OS of 13.1 months (95% CI: 10.9–15.9). Serum samples used for metabolomic study were collected before initiating treatments, and were stored at a temperature of −80 °C until the NMR measurements were performed. The study protocol was reviewed and approved by the local Ethics Committee (CEIIAV: Comitato etico IRST IRCCS AVR). Study number IRST B041 protocol number 5482/v.1 intern code: L3P1192. All patients provided written, informed consent.

### 4.2. Sample Preparation and NMR Measurements

Serum samples (200 μL) were processed according to standard procedures for NMR metabolomics measurement [8,9,54]. Briefly, prior to NMR analysis, sera were thawed at room temperature, and an aliquot of 200 μL was added of 400 μL of saline buffer solution (NaCl 0.9%, 50 mM sodium phosphate buffer in D_2_O containing TSP 0.05% wt for chemical shift calibration, pH 7.4) to minimize the variation in pH and transferred in a 5 mm NMR tube [54,55,56]. The NMR experiments were recorded on a Bruker Avance III NMR spectrometer (Bruker, Ettlingen, Germany), operating at 600.13 MHz for ^1^H observation, equipped with a TCI cryoprobe (Triple Resonance inverse Cryoprobe), incorporating a z-axis gradient coil and automatic tuning-matching (Supplementary: Section S1).

### 4.3. NMR Data Processing and Statistical Analyses

The CPMG spectra were processed and multivariate statistical analyses (unsupervised principal component analysis, PCA and the supervised partial least squares and orthogonal partial least squares discriminant analyses, PLS-DA and OPLS-DA), together with *K*(Cohen’s coefficient) according to the Naïve–Bayes classification, were performed using SIMCA 14 (Sartorius Stedim Biotech, Umeå, Sweden) [57,58,59] and WEKA 3.8.3 (University of Waikato, Hamilton, Waikato, New Zealand) softwares [60,61] (Supplementary: Section S2). Relevant metabolites identified by discriminant loadings in the OPLS-DA S-line plot were successively quantified by analyzing the integrals of selected distinctive unbiased NMR signals (Amix 3.9.14, Bruker Biospin, Italy). Results, represented as mean intensities and standard deviation (SD) of the selected NMR peaks, were validated by a univariate t-test, using the free MetaboAnalyst software [15]. The level of statistical significance was calculated at least at *p*-values < 0.05 with 95% confidence level. Finally, the Metabolic Pathway Analysis was performed, using as the input matrix the discriminant metabolites previously quantified by selected distinctive unbiased NMR signals [62,63]. To examine the association between the serum level of metabolites and disease free survival (DFS) for EAR and overall survival (OS) for ADV HCC patients, Kaplan–Meier survival curves were compared using the log-rank test. 

Statistical analysis of OS data was made by the MedCalc package (MedCalc^®^ version 16.8.4). X-tile 3.6.1 software (Yale University, New Haven, CT, USA) was used to determine the cutoff value for baseline levels. An estimation of the serum absolute concentration value for the corresponding metabolite identified by the Kaplan–Meier survival curves was also calculated by the standard-addition method (Supplementary: Section S3) [18].

## 5. Conclusions

The application of non-targeted serum metabolomics profiling provides early diagnostic differentiation and insight into the pathological mechanisms underlying HCC progression. We found that ^1^H-NMR metabolomics profiling could discriminate early from advanced HCC patients. Orthogonal partial least squares discriminant analysis (OPLS-DA) showed significant changes in serum glucose, lactate, lipids and several amino acids, between advanced and early HCC patients. Altogether, our results might indicate a shift of liver metabolism, in ADV HCC, toward the utilization of alternative metabolite sources to support the plastic and energetic demands of cancer cells. If the metabolic shift is a consequence of decreased glucose metabolism or increased amino acid demands remains to be investigated. On the other hand, it is well known that cachexia, causing ongoing muscle loss, is an event that accompanies cancer [64]. Finally, this study identifies a range of possible markers, whose serum levels might be indicative of HCC prognosis. Moreover, considering the high percentage of subjects with extrahepatic diseases in the ADV HCC group, these results should be validated by a larger cohort of samples to test the clinical validity of characteristic metabolites-derived classification models.

## Figures and Tables

**Table 1:** Patient’s characteristics and etiology.

Patient’s Characteristics	Patients Recommended to Radiofrequency (Early Stage) (n = 28)	Patients Recommended to Sorafenib (Advanced Stage) (n = 36)
**Median age (range)**	65 (38–86)	70 (67–71)
Gender		
Male	25 (89.3%)	32 (88.7%)
Female	3 (10.7%)	4 (11.3%)
**Diabetes**		
Yes	6 (21.4%)	15 (41.7%)
No	22 (78.6%)	21 (58.3%)
**Metformin treatment**		
Yes	4 (14.3%)	9 (25%)
No	24 (85.7%)	27 (75%)
**Etiology**		
HCV	13 (46.4%)	16 (44.4%)
HBV	4 (14.3%)	5 (13.9%)
NASH	2 (7.1%)	8 (22.2%)
Others	9 (32.1%)	7 (19.5%)
**BCLC stage**		
0/A	28 (100%)	0 (0%)
B	0 (0%)	16 (44.4%)
C	0 (0%)	20 (55.6%)
**Child pugh**		
A	25 (89.3%)	32 (88.9%)
B	3 (10.7%)	4 (11.1%)
**ECOG**		
0	28 (100%)	27 (75.0%)
>0	0 (0%)	9 (25.0%)
**Extrahepatic disease**		
Yes	0 (0%)	19 (52.8%)
No	28 (100%)	17 (47.2%)
**Portal Vein Thrombosis**		
Yes	0 (0%)	13 (26.1%)
No	28 (100%)	23 (63.9%)

**Abbreviations**: BCLC = Barcelona Clinic Liver Cancer; ECOG = Eastern Cooperative Oncology Group; HBV = Hepatitis B virus; HCV = Hepatitis C Virus; NASH = non-alcoholic steatohepatitis.

**Table 2:** Quantitative comparison of serum metabolites from ADV and EAR HCC patients.

Metabolite	Chemical Shift (ppm)	ADV Integrals (Mean ± SD)	EAR Integrals (Mean ± SD)	Ratio ADV/EAR	*p*-Value
**Alanine**	1.49	9.40 × 10^−3^ ± 3.61 × 10^-3^	1.55 × 10^−2^ ± 5.74 × 10^−3^	0.6	2.69 × 10^−6^
**Glycine**	3.60	2.42 × 10^−2^ ± 7.80 × 10^−3^	1.42 × 10^−2^ ± 8.85 × 10^−3^	1.7	9.94 × 10^−6^
**Glutamine**	2.47	8.23 × 10^−3^ ± 3.36 × 10^−3^	1.21 × 10^−2^ ± 3.52 × 10^−3^	0.7	1.22 × 10^−5^
**β-Glucose**	4.66	2.41 × 10^−2^ ± 9.20 × 10^−3^	1.29 × 10^−2^ ± 9.85 × 10^−3^	1.9	1.47 × 10^−5^
**α-Glucose**	5.25	1.75 × 10^−2^ ± 6.42 × 10^−3^	9.56 × 10^−3^ ± 7.31 × 10^−3^	1.8	2.11 × 10^−5^
**Galactose**	3.94	1.97 × 10^−2^ ± 5.66 × 10^−3^	1.28 × 10^−2^ ± 6.58 × 10^−3^	1.5	2.83 × 10^−5^
**1-Methylhistidine**	7.78	1.67 × 10^−4^ ± 8.25 × 10^−4^	8.91 × 10^−4^ ± 3.56 × 10^−4^	0.2	4.49 × 10^−5^
**Lactate**	1.34	1.25 × 10^−1^ ± 7.70 × 10^−2^	2.15 × 10^−1^ ± 1.21 × 10^−1^	0.6	9.08 × 10^−5^
**Lysine**	1.74	4.08 × 10^−4^ ± 5.87 × 10^−4^	1.07 × 10^−3^ ± 6.74 × 10^−4^	0.4	1.92 × 10^−4^
**N-acetylglycoproteins**	2.06	2.85 × 10^−2^ ± 7.64 × 10^−3^	2.24 × 10^−2^ ± 4.88 × 10^−3^	1.3	4.39 × 10^−4^
**Valine**	1.04	1.07 × 10^−2^ ± 2.77 × 10^−3^	1.27 × 10^−2^ ± 2.37 × 10^−3^	0.8	3.11 × 10^−3^

The selected Nuclear Magnetic Resonance (NMR) peaks (chemical shifts in the second column) determined in the serum ^1^H NMR spectra for each group, were used for the quantification of metabolites, reported as mean and relative standard deviation. Results were validated by the univariate t-test, with an adjusted *p*-value cut-off of 0.05.

**Table 3:** Metabolic Pathway Analysis for serum metabolites of ADV and EAR HCC patients.

Pathway Name	Matched Metabolites	Raw *p* (*10^−6^)	= −log(p)	Holm Adjust (*10^−5^)	FDR (*10^−5^)	Impact
Alanine, aspartate and glutamate metabolism	alanine, glutamine (2/24)	0.39	14.76	1.2	0.48	0.26401
Glycine, serine and threonine metabolism	glycine (1/48)	9.94	11.52	23.2	1.99	0.18774
Lysine degradation	lysine, glycine (2/47)	9.66	11.55	23.2	1.99	0.14675
Aminoacyl-tRNA biosynthesis	glutamine, glycine, valine, alanine, lysine (5/75)	0.45	14.62	1.3	0.48	0.05634
Amino sugar and nucleotide sugar metabolism	N-acetyl-d-glucosamine, α-glucose (2/88)	0.08	16.37	0.3	0.25	0.01122
Pyruvate metabolism	lactate (1/32)	0.59	7.44	328	64.80	0.13756
Lysine biosynthesis	lysine (1/32)	94.80	9.26	75.8	11.70	0.09993
Taurine and hypotaurine metabolism	alanine (1/20)	2.69	12.83	7.3	1.07	0.03237

Total number of compounds involved in each pathway and metabolites matched from the uploaded data; *p* is the original *p*-value calculated from the enrichment analysis; the impact is the pathway impact value calculated from pathway topology analysis.

## Supplementary Materials

The following are available online at https://www.mdpi.com/2072-6694/12/1/241/s1. Figure S1: t[2]/t[3] PCA scoreplot (the first three principal components explained 78.5% of the total variance (R2X= 0.785, Q2=0.698, t[1]= R^2^X= 0.597, Q^2^=0.549, t[2]= R^2^X= 0.116, Q^2^=0.186, t[3]=R^2^X= 0.0723, Q^2^=0.177). (1: hcv, chronic hepatitis C; 2: hbv, chronic hepatitis B; 3: NASH, nonalcoholic steatohepatitis; 4: others)., Figure S2: t[1]/t[2] PCA scoreplot (the first two principal components explained 67% of the total variance (R^2^X= 0.675, Q^2^=0.55, t[1]= R^2^X= 0.51, Q^2^=0.42, t[2]= R^2^X= 0.17, Q^2^=0.22; 1: extrahepatic; 0: no extrahepatic diseases for ADV HCC patients)., Table S1: Classifier Output from Weka analysis, according to Naïve–Bayes classification (Software WEKA 3.8.3, University of Waikato New Zealand), Section S1: NMR measurements, Section S2: NMR data processing and multivariate statistical analyses, Section S3: Tyrosine measure by standard-addition method in the ^1^H NMR spectrum.
