# Supplementary material for: 1H-NMR Based Serum Metabolomics Highlights Different Specific Biomarkers between Early and Advanced Hepatocellular Carcinoma Stages
Source: Cancers (Basel). 2020 Jan 18;12(1):241. doi: 10.3390/cancers12010241 (PMC7016798; doi:10.3390/cancers12010241)
Supplement: Supplementary file 1 [file cancers-12-00241-s001.pdf]

Supplementary Materials

^{1}H-NMR Based Serum Metabolomics Highlights Different Specific Biomarkers between Early and Advanced Hepatocellular Carcinoma Stages

Andrea Casadei-Gardini, Laura Del Coco, Giorgia Marisi, Fabio Conti, Giulia Rovesti, Paola Ulivi, Matteo Canale, Giovanni Luca Frassineti, Francesco Giuseppe Foschi, Serena Longo, Francesco P. Fanizzi and Anna M. Giudetti

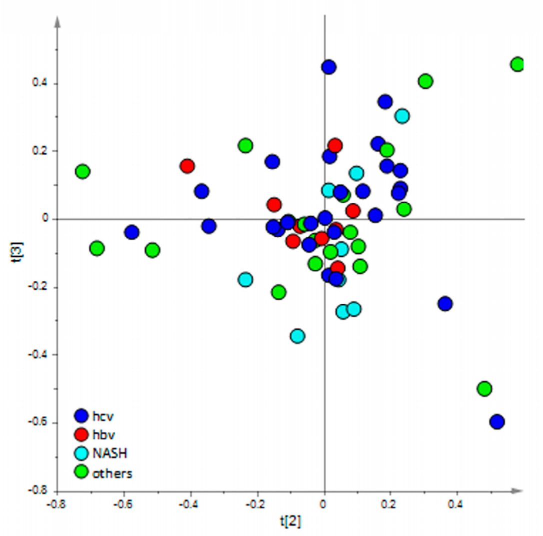

Figure S1. t[2]/t[3] PCA scoreplot (the first three principal components explained 78.5% of the total variance (R^2X= 0.785, Q^2=0.698, t[1]= R^2X= 0.597, Q^2=0.549, t[2]= R^2X= 0.116, Q^2=0.186, t[3]=R^2X= 0.0723, Q^2=0.177). (1: hcv, chronic hepatitis C; 2: hbv, chronic hepatitis B; 3: NASH, nonalcoholic steatohepatitis; 4: others).

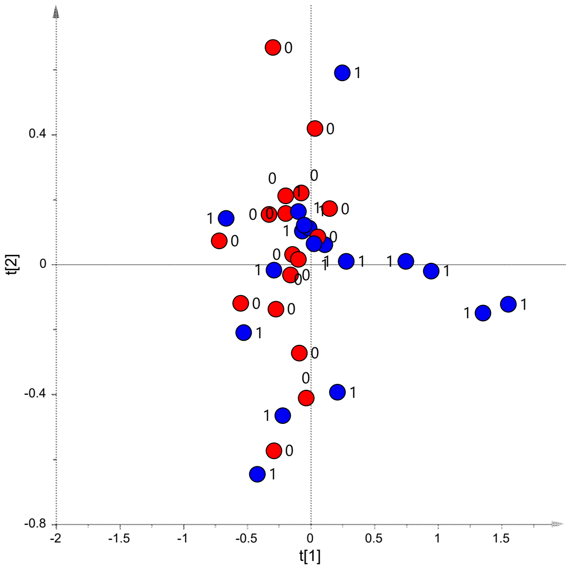

Figure S2. t[1]/t[2] PCA scoreplot (the first two principal components explained 67% of the total variance (R^2X= 0.675, Q^2=0.55, t[1]= R^2X= 0.51, Q^2=0.42, t[2]= R^2X= 0.17, Q^2=0.22; 1: extrahepatic; 0: no extrahepatic diseases for ADV HCC patients).

Section S1. NMR Measurements

Experiments were acquired at 300 K in automation mode after loading each sample on a Bruker Automatic Sample Changer, interfaced with the software IconNMR (Bruker). Measurements were repeated once in random order after completion of the first entire set. For each sample, a standard 1D ^{1}H one-dimensional spectrum with pre-saturation and composite pulse for selection (ZGCPPR Bruker standard pulse sequence) and Carr-Purcell-Meiboom-Gill (CMPG) spin-echo sequence were acquired with 32 transients, 16 dummy scans, 5 s relaxation delay, size of FID (free induction decay) of 64 K data points, spectral width of 12,019.230 Hz (20.0276 ppm), an acquisition time of 1.36 s, a total spin-spin relaxation delay of 1.2 ms, and solvent signal saturation during the relaxation delay. The resulting FIDs were multiplied by an exponential weighting function corresponding to a line broadening of 0.3 Hz before Fourier transformation, automated phasing, and baseline correction. Moreover, 2D NMR spectra (^{1}H-^{1}H J-resolved, ^{1}H-^{1}H COSY Correlation Spectroscopy, ^{1}H-^{13}C HSQC Heteronuclear Single Quantum Correlation and ^{1}H-^{13}C HMBC Heteronuclear Multiple Bond Correlation) were also randomly acquired for assignment purpose, and by comparison with published data and public databases [1-4]. The NMR spectra were processed using Topspin 3.6.1 and Amix 3.9.13 (Bruker, Biospin, Italy), both for simultaneous visual inspection and the successive bucketing process for multivariate statistical analyses.

Section S2. NMR Data Processing and Multivariate Statistical Analyses

The bucketing pre-processing procedure was applied on the CMPG spectra, covering the range 9.0-0.5 ppm, with the exclusion of the spectral region between 5.10-4.7 ppm (containing the residual peak from the suppressed water resonance). Each spectrum was segmented using the simple rectangular bucket option of the AMIX software (Bruker Biospin) in fixed rectangular regions (buckets) of 0.04 ppm width and successively integrated. The total sum normalization was applied to reduce small differences due to sample concentration and/or experimental conditions among samples [5]. The data set (bucket table) resulted in a matrix, made of 204 variables, corresponding to the bucketed ^{1}H NMR spectra values (in columns), measured for each sample (in rows). Multivariate statistical analysis (unsupervised principal component analysis, PCA and the supervised partial least squares and orthogonal partial least squares discriminant analyses, PLS-DA and OPLS-DA) were performed to examine the intrinsic variation in the data, using SIMCA 14 software (Sartorius Stedim Biotech, Umeå, Sweden) [6-8]. The Pareto scaling procedure was applied, performed by dividing the mean-centered data by the square root of the standard deviation [9,10]. The robustness of the statistical models was tested by cross-validation default method (7-fold) and further evaluated with a permutation test (400 permutations) [8]. The quality of the statistical models (in particular the total variations in the data and the internal cross-validation) was described by $R^{2,}$ Q^2 parameters and p values (p[CV-ANOVA], 95.0% confidence level, obtained from analysis of variance testing of cross-validated predictive residuals (CV-ANOVA) [11,12]. Moreover, Naïve Bayes classification, with the confusion matrix and K (Cohen’s coefficient) for the model discrimination accuracy was performed using WEKA 3.8.3 software (University of Waikato New Zealand) [13,14].

Section S3. Tyrosine Measure by Standard-Addition Method in the ^{1}H NMR Spectrum

The standard-addition method was applied in order to evaluate an estimation of the tyrosine concentration [15], corresponding to the obtained threshold-value found by Kaplan-Meier analysis for overall survival (survival probability) in the cohort of EAR stage patients, shown in figure 4 of the manuscript. Four NMR experiments were recorded with the same parameters after successive additions (at known concentration) of standard tyrosine solution to the same sample. Successively, a linear regression has been performed (R^2=0.95) and extrapolation of the line crossing at the negative portion of the concentration axis (x-axis) provided the concentration of tyrosine in the studied sample [15]. According to this method, the serum obtained concentration value has been calculated. The tyrosine level has been measured directly via integration of the corresponding NMR

signals at 7.21 and 6.91 ppm (as a mean value between the two integrals of signals). It should be noted that quantification of the metabolites in serum samples with the use of TSP is not recommended, for its potential protein binding capacity [16,17]. Due to the integral problems arising from the use of CPMG pulse sequence for absolute concentration rather than analyte ratios calculation [1], this procedure has been conducted on ^{1}H NMR spectra (zgcppr Bruker pulse program). A careful setting of the baseline correction around the selected signals was also performed, in order to minimize broad protein signal contribution. Nevertheless, also calculation performed using CPMG spectra, corrected according to Bharti et al. [15], gave comparable results.

Table S1. Classifier Output from Weka analysis, according to Naïve–Bayes classification (Software WEKA 3.8.3, University of Waikato New Zealand) [13,14].

```
=== Run information ===

Scheme:       weka.classifiers.bayes.NaiveBayes
Relation:     WekaExcel
Instances:    64
Attributes:   4
              Primary ID
              M32.YPredPS[1]($M32.DA("ADV,"))
              M32.YPredPS[1]($M32.DA("EAR,"))
              Label
Test mode:    evaluate on training data

=== Classifier model (full training set) ===

Naive Bayes Classifier

Correctly Classified Instances          63               98.4127 %
Incorrectly Classified Instances         1                1.5873 %
Kappa statistic                          0.968
Mean absolute error                      0.0183
Root mean squared error                  0.0849
Relative absolute error                  3.7003 %
Root relative squared error             17.0765 %
Total Number of Instances               64

=== Detailed Accuracy By Class ===

                 TP Rate   FP Rate   Precision   Recall    F-Measure   MCC       ROC Area   PRC
Area   Class
                 0,971     0,000     1,000       0,971     0,986       0,968     1,000      1,000
ADV
                 1,000     0,029     0,966       1,000     0,982       0,968     1,000      1,000
EAR
Weighted Avg.    0,984     0,013     0,985       0,984     0,984       0,968     1,000      1,000

=== Confusion Matrix ===

  a  b   <-- classified as
 35  1 |  a = ADV
  0 28 |  b = EAR
```

References

1. Kostidis, S.; Addie, R.D.; Morreau, H.; Mayboroda, O.A.; Giera, M. Quantitative NMR analysis of intra- and extracellular metabolism of mammalian cells: A tutorial. *Analytica Chimica Acta* **2017**, *980*, 1–24.
2. Nicholson, J.K.; Foxall, P.J.; Spraul, M.; Farrant, R.D.; Lindon, J.C. 750 MHz ^{1}H and ^{1}H-^{13}C NMR spectroscopy of human blood plasma. *Analytical Chemistry* **1995**, *67*, 793–811.
3. Ellinger, J.J.; Chylla, R.A.; Ulrich, E.L.; Markley, J.L. Databases and software for NMR-based metabolomics. *Current Metabolomics* **2013**, *1*, 28–40.
4. Fan, T.W.-M. Metabolite profiling by one-and two-dimensional NMR analysis of complex mixtures. *Progress in Nuclear Magnetic Resonance Spectroscopy* **1996**, *28*, 161–219.
5. Vu, T.; Riekeberg, E.; Qiu, Y.; Powers, R. Comparing normalization methods and the impact of noise. *Metabolomics* **2018**, *14*, 108.
6. Eastment, H.; Krzanowski, W. Cross-validatory choice of the number of components from a principal component analysis. *Technometrics* **1982**, *24*, 73–77.

7. Trygg, J.; Wold, S. Orthogonal projections to latent structures (O-PLS). *Journal of Chemometrics: A Journal of the Chemometrics Society* **2002**, *16*, 119–128.
8. Bro, R.; Kjeldahl, K.; Smilde, A.; Kiers, H. Cross-validation of component models: A critical look at current methods. *Analytical and Bioanalytical Chemistry* **2008**, *390*, 1241–1251.
9. van den Berg, R.A.; Hoefsloot, H.C.; Westerhuis, J.A.; Smilde, A.K.; van der Werf, M.J. Centering, scaling, and transformations: Improving the biological information content of metabolomics data. *BMC Genomics* **2006**, *7*, 142.
10. Antonelli, J.; Claggett, B.L.; Henglin, M.; Kim, A.; Ovsak, G.; Kim, N.; Deng, K.; Rao, K.; Tyagi, O.; Watrous, J.D. Statistical workflow for feature selection in human metabolomics data. *Metabolites* **2019**, *9*, 143.
11. Del Coco, L.; Felline, S.; Girelli, C.; Angilè, F.; Magliozzi, L.; Almada, F.; D'Aniello, B.; Mollo, E.; Terlizzi, A.; Fanizzi, F. ^{1}H NMR Spectroscopy and MVA to Evaluate the Effects of Caulerpin-Based Diet on Diplodus sargus Lipid Profiles. *Marine Drugs* **2018**, *16*, 390.
12. Del Coco, L.; Vergara, D.; De Matteis, S.; Mensà, E.; Sabbatinelli, J.; Prattichizzo, F.; Bonfigli, A.R.; Storci, G.; Bravaccini, S.; Pirini, F. NMR-Based Metabolomic Approach Tracks Potential Serum Biomarkers of Disease Progression in Patients with Type 2 Diabetes Mellitus. *Journal of Clinical Medicine* **2019**, *8*, 720.
13. Girelli, C.R.; Del Coco, L.; Fanizzi, F.P. ^{1}H NMR spectroscopy and multivariate analysis as possible tool to assess cultivars, from specific geographical areas, in EVOOs. *European Journal of Lipid Science and Technology* **2015**.
14. Consonni, R.; Cagliani, L.; Benevelli, F.; Spraul, M.; Humpfer, E.; Stocchero, M. NMR and chemometric methods: A powerful combination for characterization of balsamic and traditional balsamic vinegar of Modena. *Analytica Chimica Acta* **2008**, *611*, 31–40.
15. Bharti, S.K.; Roy, R. Quantitative ^{1}H NMR spectroscopy. *TrAC Trends in Analytical Chemistry* **2012**, *35*, 5–26.
16. Liu, Y.-Y.; Yang, Z.-X.; Ma, L.-M.; Wen, X.-Q.; Ji, H.-L.; Li, K. ^{1}H-NMR spectroscopy identifies potential biomarkers in serum metabolomic signatures for early stage esophageal squamous cell carcinoma. *PeerJ* **2019**, *7*, e8151.
17. Shimizu, A.; Ikeguchi, M.; Sugai, S. Appropriateness of DSS and TSP as internal references for 1 H NMR studies of molten globule proteins in aqueous media. *Journal of Biomolecular NMR* **1994**, *4*, 859–862.

© 2020 by the authors. Licensee MDPI, Basel, Switzerland. This article is an open access article distributed under the terms and conditions of the Creative Commons Attribution (CC BY) license (http://creativecommons.org/licenses/by/4.0/).
